# Marked deterioration in the quality of life of patients with idiopathic pulmonary fibrosis during the last two years of life

**DOI:** 10.1186/s12890-018-0738-x

**Published:** 2018-11-20

**Authors:** K. Rajala, J. T. Lehto, E. Sutinen, H. Kautiainen, M. Myllärniemi, T. Saarto

**Affiliations:** 1 Department of Palliative Care, Comprehensive Cancer Center,, Helsinki University Hospital, Paciuksenkatu 21, Po BOX 180, FI-00290 Helsinki, Finland; 2 Faculty of Medicine, University of Helsinki, Helsinki, Finland; 3 Department of Oncology, Palliative Care Unit, Tampere University Hospital and Faculty of Medicine and Life Sciences, University of Tampere, Tampere, Finland; 4 Faculty of Medicine, University of Helsinki, Helsinki, Finland; 5 Primary Health Care Unit, Kuopio University Hospital, Finland and Folkhälsan Research Center, Helsinki, Finland; 6 University of Helsinki and Helsinki University Hospital, Heart and Lung Center, Department of Pulmonary Medicine, Helsinki, Finland; 7 Helsinki University Hospital, Comprehensive Cancer Center, Department of Palliative Care and Faculty of Medicine, University of Helsinki, Helsinki, Finland

**Keywords:** Idiopathic pulmonary fibrosis, Palliative care, Health related quality of life, Symptoms

## Abstract

**Background:**

Idiopathic pulmonary fibrosis (IPF) is a chronic disease with a high symptom burden and poor survival that influences patients’ health-related quality of life (HRQOL). We aimed to evaluate IPF patients’ symptoms and HRQOL in a well-documented clinical cohort during their last two years of life.

**Methods:**

In April 2015, we sent the Modified Medical Research Council Dyspnea Scale (MMRC), the modified Edmonton Symptom Assessment Scale (ESAS) and a self-rating HRQOL questionnaire (RAND-36) to 300 IPF patients, of which 247 (82%) responded. Thereafter, follow-up questionnaires were sent every six months for two years.

**Results:**

Ninety-two patients died by August 2017. Among these patients, HRQOL was found to be considerably low already two years before death. The most prominent declines in HRQOL occurred in physical function, vitality, emotional role and social functioning (*p* < 0.001). The proportion of patients with MMRC scores ≥3 increased near death. Breathlessness and fatigue were the most severe symptoms. Symptom severity for the following symptoms increased significantly and reached the highest mean scores during the last six months of life (numeric rating scale/standard deviation): breathlessness (7.1/2.8), tiredness (7.0/2.3), dry mouth (6.0/3.0), cough (5.8/2.9), and pain with movement (5.0/3.5).

**Conclusions:**

To our knowledge this is the first study demonstrating, that IPF patients experience remarkably low HRQOL already two years before death, especially regarding physical role. In addition, they suffer from severe breathlessness and fatigue. Furthermore, physical, social and emotional wellbeing deteriorate, and symptom burden increases near death. Regular symptom and HRQOL measurements are essential to assess palliative care needs in patients with IPF.

**Electronic supplementary material:**

The online version of this article (10.1186/s12890-018-0738-x) contains supplementary material, which is available to authorized users.

## Background

Idiopathic pulmonary fibrosis (IPF) is a chronic disease with high morbidity and poor survival [1–4]. It occurs mainly in older adults, but the etiology of this progressive disease is still unknown [1]. Although the disease trajectory of IPF is variable, for many patients with IPF, survival is worse than many common malignancies. This necessitates early integration of palliative care to improve patients’ quality of life (QOL) and to relieve symptoms in addition to disease-specific pharmacological treatment and lung transplant assessment [5–9].

Existing studies have shown low health-related quality of life (HRQOL) in IPF patients. However, only few of them were prospective longitudinal studies, and most were relatively small in terms of sample size or were concentrated on pharmacological treatment [10–12]. IPF patients have been shown to suffer from lower HRQOL in real-life studies than in clinical studies [12, 13].

IPF patients suffer from many difficult symptoms, of which breathlessness and cough are the most common ones [8, 10, 14–20]. In addition, a substantial proportion of patients report anxiety, depression and pain [10, 21–26].

Dyspnea is a major contributor to HRQOL, and decreased HRQOL is associated with higher mortality [27, 28]. In a prospective Australian longitudinal registry study, impaired HRQOL was related to frequent respiratory hospitalizations and higher mortality [27]. However, to our knowledge, no previous studies have reported changes in HRQOL and symptom burden in connection with forthcoming death.

This study aimed to investigate IPF patients’ HRQOL and symptom burden during the last two years of life in a prospective longitudinal follow-up study to recognise needs for palliative care and end-of-life care planning and to characterise their symptom burden in a unique follow-up setting.

## Materials and methods

### Study population

The FinnishIPF study is a national prospective clinical registry of IPF patients that was established in 2012. IPF diagnosis is based on the ATS/ERS 2011/2015 criteria [1, 6]. Nearly all Finnish IPF patients are initially evaluated at public university and central hospitals. Patients from these specialist centres with informed consent are included in the FinnishIPF registry, which consists of approximately 76% of all Finnish IPF patients [2]. Currently, the registry contains data from over 700 IPF patients.

All 300 patients registered in the FinnishIPF study in April 2015 were asked to participate in this substudy by sending an informed consent form together with the questionnaires. Those who did not respond within two weeks were called and reminded. Of the 300 registered patients, 247 (82%) provided informed consent for this substudy, answered the first questionnaire and were included in this study. Subsequently, the same questionnaire was sent to the patients five times at six months intervals until August 2017.

### Data collection and questionnaires

Disease and sociodemographic characteristics were collected from patient records and with a separate questionnaire (Additional file 1). These included the date of birth, sex, age, marital status, education, living conditions, physical activity level, the need for assistance in daily activities, the date of IPF diagnosis, smoking status, and comorbidities. Patients were asked the frequency of leisure time physical exercise that causes breathlessness and sweating for a minimum 30 min during the preceding six months. Death certificates were acquired from the “National Authority for Collecting and Compiling Statistics on Various Fields of Society and Economy”.

The questionnaires regarding HRQOL and symptoms were the RAND 36-Item Health Survey (RAND-36), the Modified Medical Research Council Dyspnea Scale (MMRC), and the modified Edmonton Symptom Assessment Scale (ESAS).

RAND-36 [29] is a general QOL measurement tool with existing Finnish general population reference values [30]. RAND-36 is similar to the previously IPF-validated short-Form-36 [30–32]. RAND-36 is divided into eight health concepts [29, 30]. Concepts are scored on a scale from 1 to 100, where a lower score indicates a worse HRQOL during the past four weeks [29, 30]. The concepts are as follows: “general health” (five questions), “vitality” (four questions regarding energy level and tiredness), “bodily pain” (two questions), “physical functioning” (ten questions regarding the ability to take care of personal hygiene and the ability to move and exercise), “physical role” (four questions regarding role limitations due to physical health), “mental health” (five questions regarding mood, depression and anxiety), “emotional role” (three questions regarding role limitations due to emotional problems), and “social functioning” (two questions) [29, 30].

The self-rated MMRC measures the degree of disability that breathlessness causes during day-to-day activities on a scale from 0 to 4, in which 0 indicates no breathlessness except during strenuous exercise, 1 indicates shortness of breath when walking up a slight hill or hurrying on a level, 2 indicates walking slower than people of same age on a level because of breathlessness or needing to stop to for breath when walking at one’s own pace on a level, 3 indicates needing to stop for breath after a few minutes when walking on a level or after walking approximately 100 m, and 4 indicates that the patient is too breathless to leave the house or is breathless when dressing or undressing [33, 34].

The ESAS is a numeric self-rating symptom-based scale that was originally developed to assess the symptoms of cancer patients [35, 36]. Different symptoms are measured on Numeric Rating Scale (NRS) from 0 (no symptoms) to 10 (the worst possible symptoms) [36–38]. In this study, we used a version including 12 symptoms (pain at rest, pain with movement, tiredness, nausea, depression, anxiety, insomnia, loss of appetite, shortness of breath, cough, constipation, dry mouth, and overall wellbeing). There is a lack of evidence to recommend cut-off points for the ESAS. However, an NRS score ≥ 4 is commonly used as a trigger for more comprehensive symptom assessment in clinical practice [39].

### Statistics and ethical aspects

The study population characteristics are presented as the means with standard deviations (SD) or as counts with percentages. Patients’ answers were grouped, according the time they answered from the aspect of death. The Kaplan-Meier method was used to estimate the cumulative mortality after the diagnosis. We used restricted cubic splines to detect a possible non-linear dependency. A nonlinear relationship between the RAND-36 domains, symptom severity, the MMRC and time before death were assessed by using 5-knot-restricted cubic spline random-effects regression models with appropriate distribution and link functions. Models included age and gender (only main effects) as covariates. A test of interaction between independent variables was performed through the MFPIgen command. The length of the distribution (months before death) of knots was located at the 5th, 27.5, 50th, 72.5, and 95th percentiles, which correspond to time before death of − 22, − 15, − 9, − 5 and − 1. The locations of the knots were determined by the percentiles recommended in Harrell’s publication [40].The normality of the variables was tested by using the Shapiro-Wilk test. The Finnish general population values for the eight Rand-36 domains were weighted to match the gender and age distribution of the study population, statistical analysis between our population and general population was not performed [30]. The Stata 15.0 [41] statistical package was used for the analysis.

The ethics committee of Helsinki University Central Hospital approved this study (381/13/03/01/2014). The Finnish National Institute for Health and Welfare (Dnro THL/1161/5.05.01/2012) approved the screening of hospital registries for patients with IPF. All participating patients provided written informed consent to participate to this specific study.

## Results

**Fig. 1:**
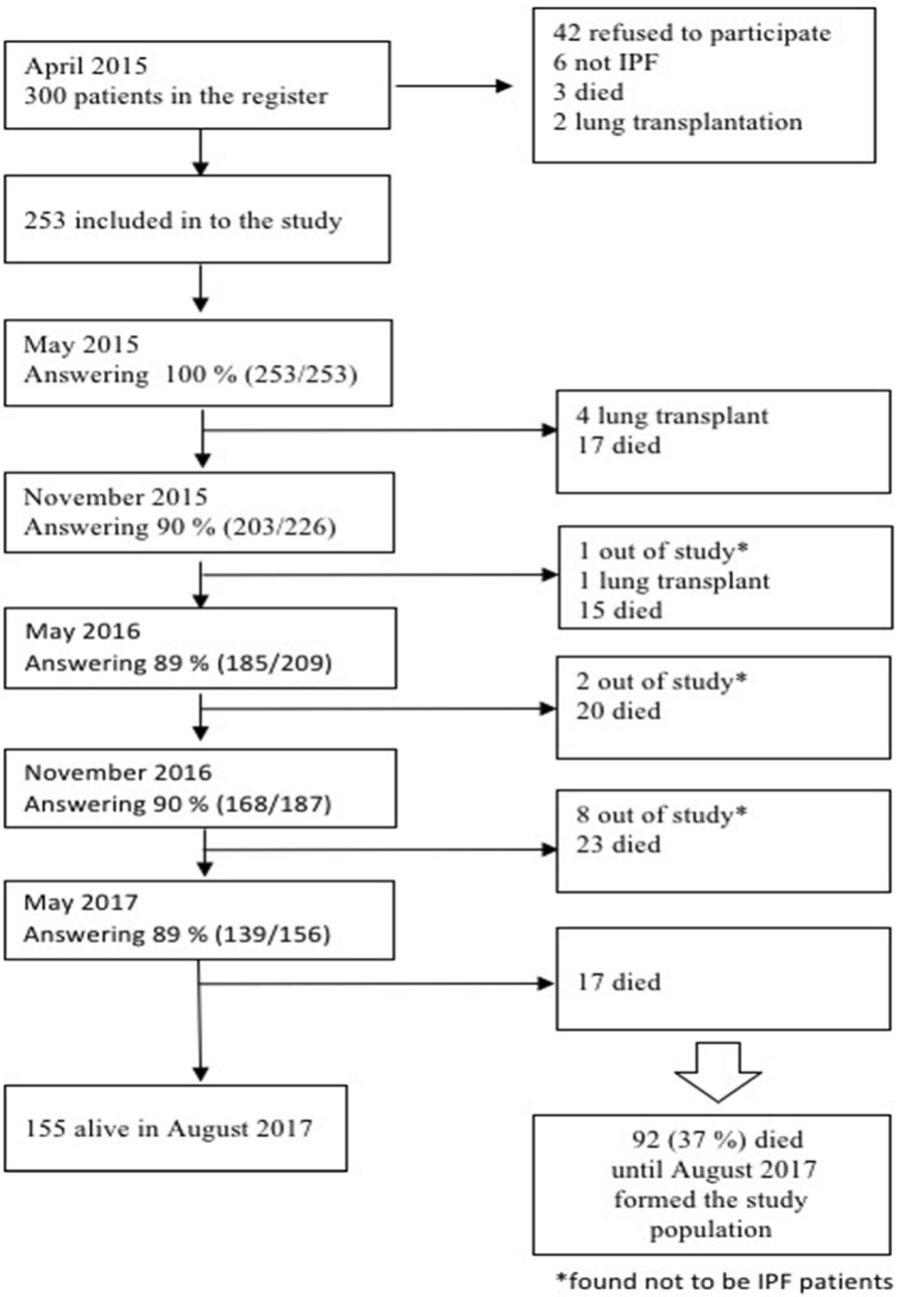
Flowchart of patient recruitment and response rate

Of the 247 patients included in the study 92 (37%) died by August 2017 and were included in our follow-up cohort (Fig. 1).

**Fig. 2:**
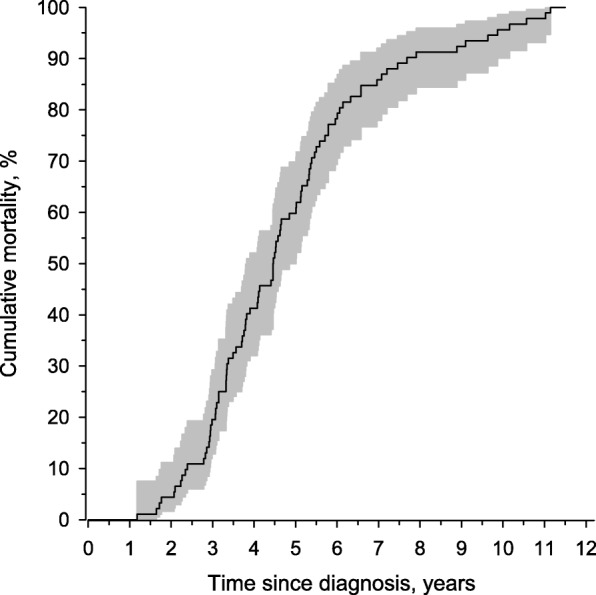
Cumulative mortality after the diagnosis of IPF. Time-point of the diagnosis is marked with 0 and 95% confidence intervals with the grey area. Kaplan-Meier method was used to estimate the cumulative mortality

The cumulative mortality of the patient cohort (*n* = 92) is presented in Fig. 2. The median overall survival was 4.4 years (IQR 3.1–5.7). Patient characteristics are shown in Table 1. A majority of the patients had comorbidities, of which cardiovascular diseases were the most common ones. None of the patients had lung cancer when entering the study, but two patients were diagnosed with lung cancer during the follow-up. Lung function measurements within the last six months of life were available in only 28 (30%) of the patients (mean FVC 2.2 l (SD 0.6), 57%). Antifibrotic medication was used by 33 (35%) of the patients.

**Table 1:** Patient characteristics

Total number of patients	92
Age, mean (range)	75 (57–92)
Males n (%)	67 (73)
Duration of IPF in years, mean (SD)	3.6 (2.3)
Education in years, mean (SD)	10 (3)
Living alone, n (%)	31 (34)
Working, n (%)	4 (4)
Smoking status, n (%)^a^	
Smoker	6 (7)
Ex-smoker	50 (54)
Never-smoker	36 (39)
FVC (litres), mean (SD)^b^	2.9 (0.8)
FVC (% of predicted), mean (SD)^b^	78 (16)
Diffusion capacity, mean(SD)^c^	54 (13)
Co-morbidities^e^, n (%)	
Hypertension	41 (45)
Coronary heart disease	35 (38)
Diabetes	24 (26)
Heart failure	23 (25)
COPD	20 (22)
Cancer	16 (17)
Asthma	8 (9)
No co-morbidities	13 (14)
Number of co-morbidities, median (range)	2 (0–7)
Place of death, n (%)^b^	
Hospital^d^	62 (67)
Home	19 (22)
Nursing home	4 (5)
Hospice	3 (3)

^a^smoking status, forced volume vital capacity (FVC) and diffusion capacity are recorded at the time of diagnosis and other factors at the time of the first questionnaire

^b^Data missing from 4 patients; ^c^ data missing from 12 patients; ^d^10 in intensive care unit; ^e^ patient-reported co-morbidities

The proportion of the patients who could not perform continuous moderate intensity physical exercise for at least 30 min during the previous six months increased from 34% 18–24 months before death to 62% during the last six months of life. Six months before death, 67% of the patients reported needing assistance with their daily activities, while 18–24 months before death this proportion was 56%.

### Health-related quality of life

**Fig. 3:**
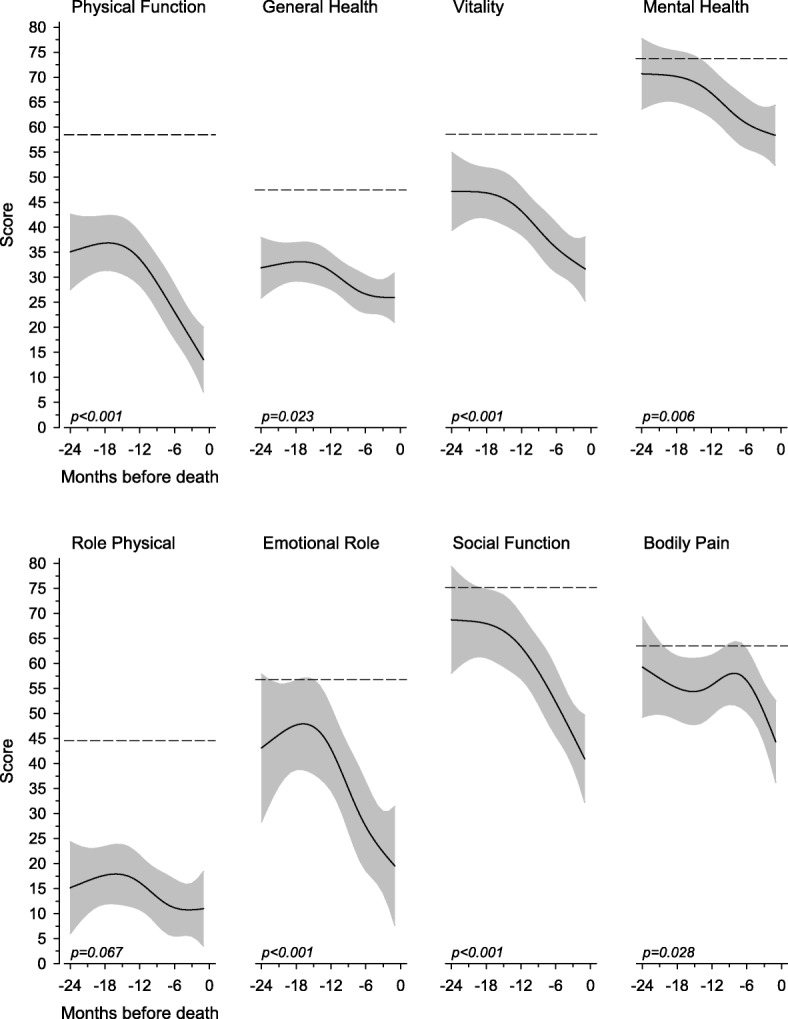
Relationships between Health-related quality of life domains (RAND-36) two years before death. The curves were derived from a 5-knot restricted cubic splines regression models. The models were adjusted for age and gender. The grey area represents 95% confidence intervals. Dashed lines mark Finnish general population levels

Figure 3 shows the changes in the different dimensions of HRQOL measured with RAND-36 during two years before death. The decline in HRQOL was highly significant in all dimensions except in physical role, which showed very low scores by 24 months before death.

### Symptoms

**Fig. 4:**
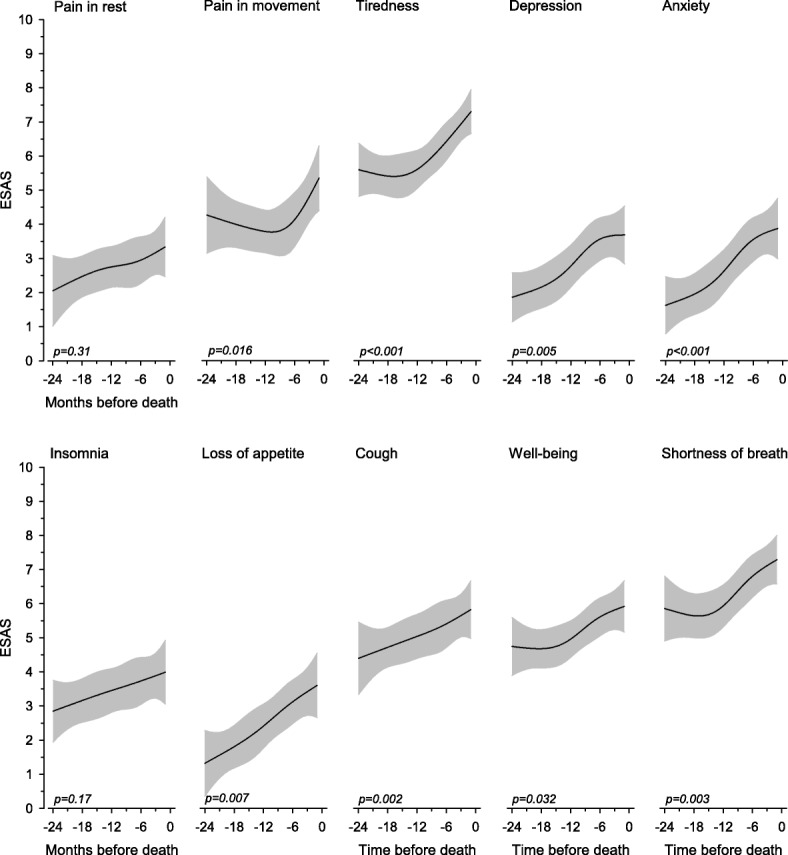
Relationships of symptom severity measured by ESAS two years before death. The curves were derived from a 5-knot restricted cubic splines regression models. The models were adjusted for age and gender. Grey area represents 95% confidence intervals. ESAS, Edmonton symptom assessment scale

The intensity change of the symptoms measured by the ESAS during the last two years of life is presented in Fig. 4. The intensity of all symptoms except pain at rest and insomnia increased significantly near death. During the last six months of life, the mean NRS scores were as follows: 7.1 (SD 2.8) for breathlessness, 7.0 (SD 2.3) for tiredness, 6.0 (SD 2.5) for wellbeing, 6.0 (SD 3.0) for dry mouth, 5.8 (SD 2.9) for cough, 5.0 (SD 3.5) for pain with movement, 3.9 (SD 3.1) for insomnia, 3.9 (SD 2.9) for anxiety, 3.8 (SD 2.9) for depression, 3.6 (SD 3.1) for constipation, 3.4 (SD 3.3) for loss of appetite, 3.1 (SD 2.8) for pain at rest and 1.8 (SD 2.5) for nausea.

**Fig. 5:**
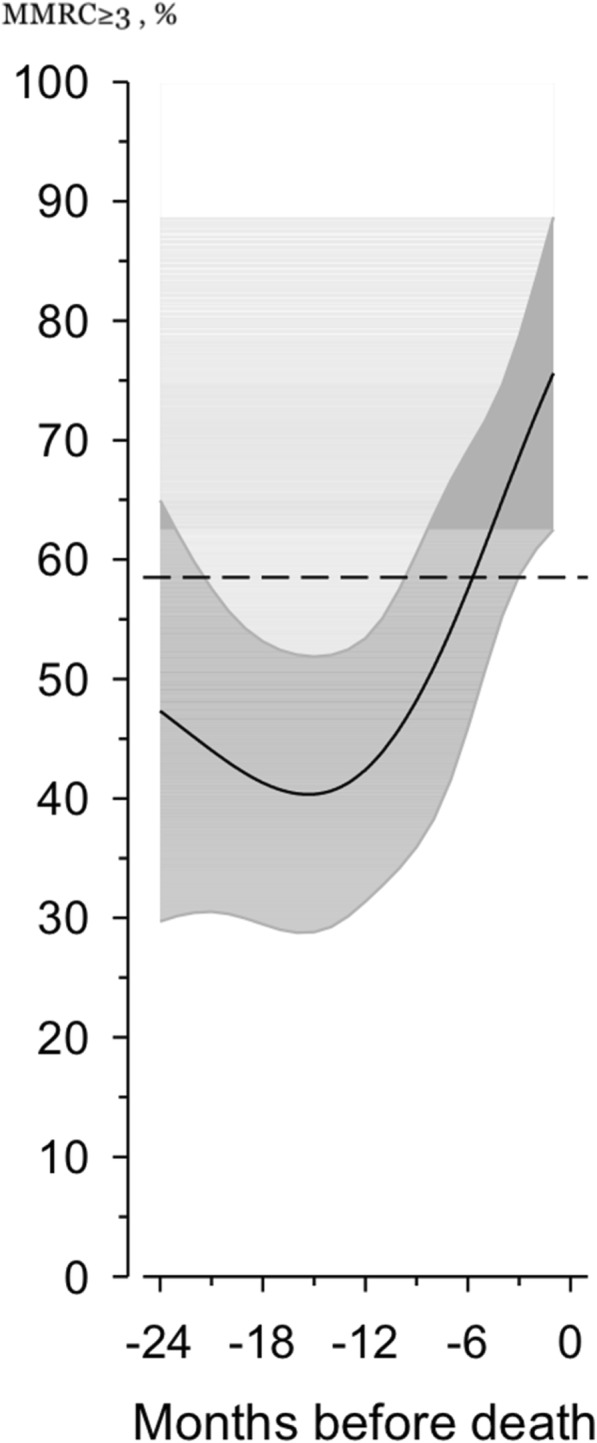
Change in the proportion of patients with MMRC score ≥ 3 during the last two years of life. The curves were derived from a 5-knot restricted cubic splines logistic regression models. The models were adjusted for age and gender. The grey area represents 95% confidence intervals

The steep change in the proportion of patients with MMRC scores ≥3 (needing to stop walking after approximately 100 m or a few minutes because of breathlessness) during the last two years of life is shown in Fig. 5.

## Discussion

In this study, we demonstrate a rapidly increasing impairment in HRQOL and escalating symptom burden in IPF patients approaching death. Low HRQOL together with severe breathlessness and fatigue were detected as early as two years before death. In addition, several dimensions of HRQOL declined further and the severity of many symptoms other than dyspnea increased during the last two years of life.

In the present study, HRQOL was considerably impaired two years prior to death in IPF patients. Physical role, i.e., role limitations due to physical health, was exceptionally low, but physical functioning, vitality and general health appeared to be below the general population level as well. Similar to our findings, an Australian registry study of 516 IPF patients reported HRQOL impairments in all domains, with the lowest score in activity, i.e., activities that cause or are limited by breathlessness [27]. The importance of decreased HRQOL was further highlighted in a recent study by Furukawa et al. that demonstrated that low HRQOL was actually an independent prognostic factor [28].

Although previous studies have demonstrated low HRQOL in IPF patients [10–12], none of them focused on the HRQOL from the aspect of approaching death in a follow-up setting. The uniqueness of this study stems from the continuing follow-up until death, and the subsequent finding of a salient decline in HRQOL during the last two years of life that was intensified near death. Deterioration was identified in all domains except physical role, which was already remarkably low two years before death. The most integral impairment was in physical, social and emotional functioning and vitality. In the Australian registry study, approximately one-third (38%) of the IPF patients experienced clinically important differences in the decline of HRQOL during 12 months [27]. However, in that study, no HRQOL data was available from the period preceding death [27].

In lung cancer, functional concerns relating to physical movement or functioning predominate patients’ symptom burden throughout the disease course and have a negative impact on HRQOL [42]. In addition, the severity of symptoms escalates, and the number of severe symptoms increases during the last three months of life [42–44]. A steep decline in HRQOL at the end-of-life is typical in patients dying of cancer compared with other terminally ill patients [45, 46]. In COPD patients, HRQOL gradually declines over time without a steeper decline at the end-of-life [47]. Our data imply that patients with IPF experience gradual impairments in HRQOL comparable to COPD patients but suffer from a pronounced, rapid deterioration in HRQOL during the last year of life, more closely resembling the disease trajectory of cancer.

Our results corroborate earlier findings on dyspnea and cough as the most severe symptoms in IPF patients regardless of the disease phase [10, 21, 27, 48]. The intensity of dyspnea increased during the follow-up, being one of the most severe symptoms before death. In a previous IPF registry study, dyspnea yielded the strongest association with impaired HRQOL accounting for 71% of the variation in HRQOL [27]. In addition, exertion dyspnoea measured by the MMRC has been shown to correlate to HRQOL and symptom burden [21, 49].

In addition to dyspnea, other activity-limiting symptoms such as fatigue and pain in movement were among the most severe symptoms in our study. These activity-limiting symptoms can lead to physical inactivity and functional impairment, triggering a vicious circle with worse HRQOL. The intensity of depression and anxiety were, however, relatively mild two years before death, although these symptoms increased thereafter. In a recent study, depression was an independent predictor for HRQOL impairment, although it only accounted for 3.5% of the variation, whereas dyspnea accounted for 71% [27]. Our results suggest that the relief of activity-limiting symptoms together with psychosocial support may improve HRQOL in advanced IPF.

As discussed above, patients with advanced IPF, COPD and lung cancer suffer from a heavy symptom burden and deteriorating HRQOL. This calls for comprehensive symptom management and integrated palliative care concomitant with disease-modifying therapies [21, 27, 47, 50–52]. Early integrated palliative care for patients with lung cancer has shown substantial benefits, such as lower depression scores, higher HRQOL, better communication of end-of-life care preferences, less aggressive care at the end-of-life, and longer overall survival [51, 53]. Similarly, a randomised trial demonstrated better control of dyspnea and a survival benefit with integrated palliative care in patients with COPD and interstitial lung disease [54]. In addition to cancer patients, early integrated palliative care may reduce end-of-life acute care utilisation, and allow patients with IPF to die in their preferred locations [55–58]. Integrated palliative care in IPF patients seems to lower respiratory-related emergency room visits and hospitalisations and may allow more patients to die at home [55]. In this study, 67% of patients died in hospital and 11% in intensive care, which is in line with earlier findings, implying the necessity of improvements in advanced care planning and palliative care of patients with IPF [55, 59]. Our results provide insight into the most important needs of end-stage IPF patients and support the use of early-integrated palliative care, which should include symptom control beyond treatment for dyspnea and psychosocial support.

The relatively small study population limits our study, as did not having systematic follow-up data on lung function, which is at least partially due to the poor conditions of many of our patients. Our study is to our knowledge first to present follow-up data of HRQOL and symptoms for over two years before death. The strength is its real-life longitudinal design, with a unique cohort of IPF patients approaching death with an outstanding response rate, particularly considering the fact that some of the patients were probably too weak to respond during their final days or weeks of life. To our knowledge, this is the first study describing comprehensive patient-reported data on the HRQOL and symptom burden of IPF patients from the perspective of approaching death.

## Conclusion

Patients with IPF suffer from exceptionally low HRQOL together with severe breathlessness and fatigue already two years before death. In addition, physical and emotional wellbeing further deteriorates near death concurrently with escalating overall symptom burden. In clinical practice, structured measurements of HRQOL and symptoms are necessary to guide high-quality early-integrated palliative care and end-of-life planning in IPF patients.

## Additional file

Additional file 1: Disease and sociodemographic characteristics. The questionnaire used to collect disease and sociodemographic characteristics. (DOCX 35 kb)

## Abbreviations

COPD: Chronic obstructive pulmonary disease
EOL: End-of-life
ESAS: Modified Edmonton Symptom Assessment Scale
FVC: Forced vital capacity
HRQOL: Health-related quality of life
IPF: Idiopathic pulmonary fibrosis
IQR: Interquartile range
MMRC: Modified Medical Research Council Dyspnea Scale
NRS: Numeric rating scale
RAND-36: RAND 36-Item Health Survey
SD: Standard deviation
SGRQ: St Georges Respiratory Questionnaire
