# Supplementary material for: Marked deterioration in the quality of life of patients with idiopathic pulmonary fibrosis during the last two years of life
Source: BMC Pulm Med. 2018 Nov 20;18:172. doi: 10.1186/s12890-018-0738-x (PMC6247520; doi:10.1186/s12890-018-0738-x)
Supplement: Supplementary file 1 — Disease and sociodemographic characteristics. The questionnaire used to collect disease and sociodemographic characteristics. (DOCX 35 kb) [file 12890_2018_738_MOESM1_ESM.docx]

Baseline questionnaire:

Quality of life in Patients with Idiopathic Pulmonary Fibrosis (IPF)

Name:

Social security number: ___/___/___ - _______

1. Marital status, are you currently

1. married/registered partnership

2. co-habitating

3. divorced or in a legal separation

4. widowed

5. unmarried

2. Family

1. under-aged children (<17yrs of age), living at home

2. under-aged children (<17yrs of age), not living at home

3. adult children

4. no children

3. Living

1. alone

2. with spouse

3. other living arrangement, what?____________

4. Education (choose the highest education attained)

1. In the former Finnish school system

2. elementary school

3. vocational school

4. high school

5. polytechnic

6. lower academic degree

7. higher academic degree

8. licentiate

9. PhD

5. Occupation:___________________

6. If you are employed at the moment, how physically strenuous is your work? (circle the option that best describes it).

1. I mainly sit, I do not walk a lot during my workday.

2. I walk quite much, but I do not need to lift or carry heavy objects.

3. I need to walk a lot and lift heavy objects or climb stairs or walk uphill.

4. My work is heavy physical labor, and I have to lift or carry heavy loads, dig, shovel or chop, etc.

7. Currently I am…

1. working full time

2. working part time

3. retired, due to:

a. age

b. disability pension, due to this disease

c. disability pension, due to other disease

4. unemployed or suspended without pay

5. on sick leave (over four weeks)

1. due to this disease

2. due to other disease

6. not employed

7. a student

8. Type of housing

1. apartment building

1. which floor do you live in:______

2. the house has an elevator

0 no

1 yes

2. duplex or townhouse

1. one-story

2. more than one-story

3. house

1. one-story

2. more than one-story

4. assisted living facility

5. elderly care home

6. long-term institutional care

7. other housing type, specify _________

9. I have/ my co-morbidities are

1. hypertension 0 no 1 yes

2. coronary artery disease 0 no 1 yes

3. chronic obstructive pulmonary disease(COPD)

0 no 1 yes

4. asthma 0 no 1 yes

5. Have you suffered from a heart attack 0 no 1 yes

6. stroke (brain hemorrhage or infarction)

0 no 1 yes

7. osteoporosis 0 no 1 yes

8. diabetes mellitus 0 no 1 yes

9. cancer 0 no 1 yes, which?

10. heart failure 0 no 1 yes

11. psychiatric illness 0 no 1 yes

12. Parkinson’s disease 0 no 1 yes

13. Multiple sclerosis 0 no 1 yes

14. other, which?:_____________

10. How much have you exercised or strained yourself physically in your free time during the last 6 months? If there is a major difference in different seasons, mark the average.

1. I read, watch TV or do chores with do not require much physical effort.

2. I walk, cycle or exercise a minimum of four hours per week. Includes walking, fishing and hunting, light gardening etc. but not commuting.

3. In my spare time, I exercise intentionally, ie. I do running, jogging, skiing, gymnastics, swimming, ball games or strenuous gardening or other similarly strenuous activies a minimum of 3 hours per week.

11. How often have you exercised during the last 6 months, a time of minimum 30 minutes, so that you are at least little out of breath or sweaty (for example jogging, skiing, bicycling, swimming, Nordic walking, aerobics, ball games et cetera)? Choose the best option

1. I can not due to a disease or injury

2. few times or less

3. 1-3 times per month

4. once a week

5. 2-3 times per week

6. 4-5 times per week

7. over 5 times per week

12. How many minutes have you walked, cycled or used any other physically active way of transportation in your work or everyday chores daily? (means time used back and forth)

1. I do not walk or cycle daily for work or chores

2. under 15 minutes daily

3. 15 - 29 minutes daily

4. 30 - 44 minutes daily

5. 45 - 59 minutes daily

6. over one hour daily

13. I know who to contact if my disease worsens

0 no

1 yes

1. health care center emergency

2. hospital emergency

3. hospital pulmonary clinic

4. home care / hospital

5. other, what: ____________

14. Do you get regular help in your everyday chores, due to a decreased performance? For example help in shopping, housekeeping, washing et

0 no 1 yes 2 no, but I would need help

15. How often would you need the abovementioned assistance?

1. once a month or less frequently

2. 2-3 times per month
 3. once a week

4. few times a week
 5. daily

6. most of the day
 7. almost 24/7

17. I mainly get help from

1. my spouse

2. children or their spouses

3. other relatives, neighbours or friends

4. home aid

5. home nurse

6. others, who?________
